# Who Are the Regulars ?

**Published:** 1850-11

**Authors:** 


*Who are the Regulars ?* In variety of doctors, New York is beyond all England, and the distinction, if we could enumerate them all, would be multitudinous and amusing.

1. Regular graduates, M. D's., who claim to be both Physicians and Surgeons.

2. Honorary graduates, M. D's., many of whom are guiltless of any education of any sort.

3. Fictitious M. D's., who may or may not be licentiates, and yet they claim the title and wear it.

4. Licentiates of any county or state societies, who when honest, prefix the letters Dr. to their names; though many of these fictitiously assumed to be graduates.

5. Homœpaths, all of whom sign their names M. D., whether with or without authority.

6. Hydropaths, ditto.

7. Magnetic and Mesmeric doctors, some of whom claim the title.

8. Paw doctors, who profess to cure by friction with the hand.

9. Indian doctors, who profess to have knowledge of aboriginal remedies.

10. Cancer doctors, who promise to cure cancer without the knife, and who apply arsenic and other caustic plasters.

11. Seventh-son doctors, whose "*larning cum by natur.*"

12. Thompsonian doctors, who use lobelia, cayenne, and steam.

13. Natural bone setters, who cure by dislocating and reducing all the bones at pleasure.

14. Botanical doctors, who claim to reject all minerals, and rely for remedies on *roots and yarbs.*

And besides all these we have so-called oculists, and aurists, lung, liver, kidney, and urine doctors, dyspepsia doctors, and a numerous class of Lock Hospital, or secret doctors, with pile and corn doctors, *et id genus omne,* whose name is Legion ; so that surely in this great city no one need suffer without doctoring, or being doctored, in every conceivable variety.

Thus far no mention has been made of the tribe of blood doctors—and pill doctors, and panacea doctors, and bitters doctors, and consumption doctors, and the clergymen's sore throat doctors; though many of these are veritable M. D's. But our limits will not permit us to enlarge at present, though we are sensible very many of the doctors have not yet been named. Indeed, some of them, it would be a shame even to name, so shameless is their craft.

Our British brethren, however, have nothing to boast of in the way of exemption from quackery. So far from it, we are indebted to England, especially to London, for the worst examples among us; but it is fair to presume that many of them "left their country for their country's good."—*N. Y. Med. Gazette.*

